# Hybrid quasi Z source multi output converter system with performance control and real time validation for photovoltaic microgrid

**DOI:** 10.1038/s41598-026-35817-7

**Published:** 2026-01-26

**Authors:** Pooja Deori, Anish Ahmad, Abhinandan Routray

**Affiliations:** 1 Department of Electrical Engineering, Tezpur University, Tezpur, 784028 India; 2 Manipal Institute of Technology, Manipal Academy of Higher Education, Manipal, Karnataka - 576104 India

**Keywords:** Hybrid multi-output converter, Photovoltaic (PV) system, Maximum power point tracking (MPPT), Perturb and observe (P& O) algorithm, Decoupled closed-loop control, Energy science and technology, Engineering

## Abstract

This work demonstrates a real-time validation of a photovoltaic-based hybrid quasi-Z source multi-output converter system for microgrid applications. The conventional microgrid system consists of several converter systems designed to meet ac and dc power load requirements. This configuration makes the overall system bulky, increases costs, and reduces efficiency. However, in the proposed system, a hybrid concept is used to get multiple ac and dc power simultaneously in a single-stage conversion, which is a common requirement in the microgrid system. In this proposed system, photovoltaic arrays are used as input power with the perturb and observe-based maximum power point tracking algorithm to get the maximum power utilization of the PV system. Moreover, the proposed system consists of three outputs that are designed with a closed-loop control. Closed-loop control uses a new control method developed with the utilization of duty cycle and modulation index as independent control variables to control the ac and dc outputs. A detailed mathematical analysis of the proposed system is carried out, and the effectiveness of the presented control strategy is validated with simulation. Additionally, Hardware-In-Loop (HIL) validation is performed for a Typhoon HIL-based real-time emulation platform to evaluate the proposed system.

## Introduction

With the rapid growth of population and development of the world economy, energy consumption is increasing at a higher rate, leading to more usage of non-renewable energy, causing pollution and threat to the environment. To address these pressing issues, wind energy and solar photovoltaic (PV), in particular, have become prominent part of renewable energy based microgrid system due to its numerous advantages such as clean and pollution-free electricity production, short construction periods, and flexible load allocation capabilities^1^.

**Fig. 1:**
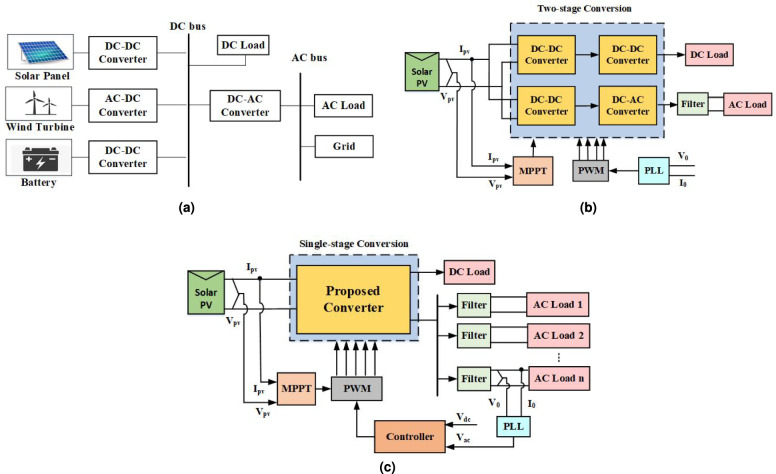
Structure of microgrid (**a**) typical microgrid, (**b**) conventional two-stage dc–ac converter system, (**c**) proposed single-stage hybrid multi-output converter system.

The increasing demand for electrical power is putting a strain on the existing transmission and generation system capabilities, which can result in frequent power outages. To address this issue, there are a number of different methods that can be used to enhance the reliability and security of the electrical power system^2–4^. Microgrid is one of the significant solutions where the power generation is closer to the consumer areas^5^. Various studies on control, stability and applications of ac, dc, and hybrid microgrid systems are found in the literature^6,7^. Recent studies have emphasized the importance of advanced control strategies and intelligent power electronic interfaces in improving the stability and dynamic performance of renewable-energy-based power systems^8^. Intelligent Maximum Power Point Tracking (MPPT)-based inverter control and dc-link voltage regulation are widely acknowledged for their critical role in maintaining overall system stability under variable operating situations, particularly in solar PV oriented grid-connected systems. Hybrid renewable configurations incorporating PV and wind resources, along with auxiliary control functionalities such as static synchronous compensator (STATCOM) operation, have also been reported to enhance multimachine power system stability and dynamic response^9–11^. Furthermore, advanced control techniques, including metaheuristic optimization and fractional-order control, have been explored to improve voltage regulation, power quality, and stability margins in grid-connected PV systems^12^. A traditional structure of microgrid is shown in Fig. 1(a), which uses multiple converter stages to supply the loads. The typical two-stage structure uses a dc-to-dc converter^13^ in the preliminary stage with boost or buck-boost nature and a voltage source inverter (VSI) in the secondary stage for conversion from dc to ac^14^ as shown in Fig. 1(b). One of the demerits of conventional two-stage architecture is the usage of several power converters resulting in increased system size and makes the system voluminous and costly. Moreover, a lot of energy is wasted in the conversion process, due to which power loss is more and the efficiency of the system is reduced. Therefore, to meet this demand, a hybrid topology of the converter must be designed which is both compact in size and capable of providing multiple hybrid outputs for different applications as illustrated in Fig. 1(c).

The single-stage structures have emerged as a solution to deal with the drawbacks associated with two-stage configurations^15^. One notable idea is to utilize the impedance-source (Z-source) network that is connected to the input side of a PV system. This Z-source network enables the converter to operate its output voltage in both buck and boost modes with its single-stage configuration enabling it to regulate and control the voltage output effectively^16^. Despite the advantages of Z-source inverters (ZSIs), some of the drawbacks of conventional ZSI are the discontinuous input current, high capacitor voltage stress and less voltage boost capability. To address this concerns, several quasi-Z source inverter (Q-ZSI) topologies are proposed in^17,18^ which has inherent limitation to input inrush current and less Z-source capacitors and inverter-bridge voltage stress. However, despite the advantages, the voltage conversion ratio of the Q-ZSI is low, prompting researchers to explore modifications and adding passive components in the impedance network^19–21^. Moreover, references^22–24^ reviews different topologies of hybrid converter based on Q-ZSI having high voltage boost factor. Nevertheless, a further issue with these converters is their inability to meet the voltage level and multiple concurrent requirements of contemporary electrical systems, including hybrid microgrids and electric cars due to the single output port. To address this concerns, several multi-output converters are proposed in^25–28^. Another class of Q-ZSI topology based hybrid converter is proposed in^29^, which comprises of two three-phase Q-ZSIs producing two multiple outputs but restricted to ac outputs only. In order to address these concerns, a hybrid converter is proposed in^30^, which can produce two dc and one ac output simultaneously with both boost and buck ability. Although the converter mentioned above can generate both ac and dc outputs, it is limited to one ac output. This is resolved in the proposed paper^31^ which generates n-number of ac and one dc simultaneous outputs and another work^32,33^, which utilize both series and parallel mode configuration with simultaneous provision of two dc outputs and multiple ac outputs. A Q-ZSI topology with MPPT control, proposed in^34,35^, aims to connect PV arrays to the utility grid. It explores operating conditions in large-scale PV plants with battery storage, but the single-stage conversion still falls short of meeting hybrid power demands, especially for residential use.

This work makes the following key contributions:

(i) The proposed work gives a comprehensive advancement at the topology, control, and system-integration levels over existing single-stage PV-fed quasi-Z-source and multiport converter architectures. Specifically, a modified quasi-Z-source multi-output converter system is developed by integrating an additional switched-capacitor branch, enabling the simultaneous realization of a regulated boost dc output and multiple ac outputs within a single power-processing stage. Furthermore, the dc output is directly extracted from the impedance network and actively regulated, rather than being treated as an auxiliary or uncontrolled port as in conventional Q-ZSI-based multiport converters.

(ii) Second, a decoupled control strategy is analytically developed, wherein the modulation index and the shoot-through duty ratio are treated as independent control variables to regulate the ac and dc outputs, respectively, subject to a practical PWM constraint. This decoupling is explicitly derived, validated under dynamic load conditions, and demonstrated to maintain independent regulation of hybrid outputs.

(iii) The proposed topology and control scheme are validated at the system level through both detailed simulation and real-time hardware-in-the-loop (HIL) testing using Typhoon HIL 402, demonstrating simultaneous dc and multi-ac power delivery suitable for hybrid microgrid applications.

## Overall system configuration of the presented solar PV system

**Fig. 2:**
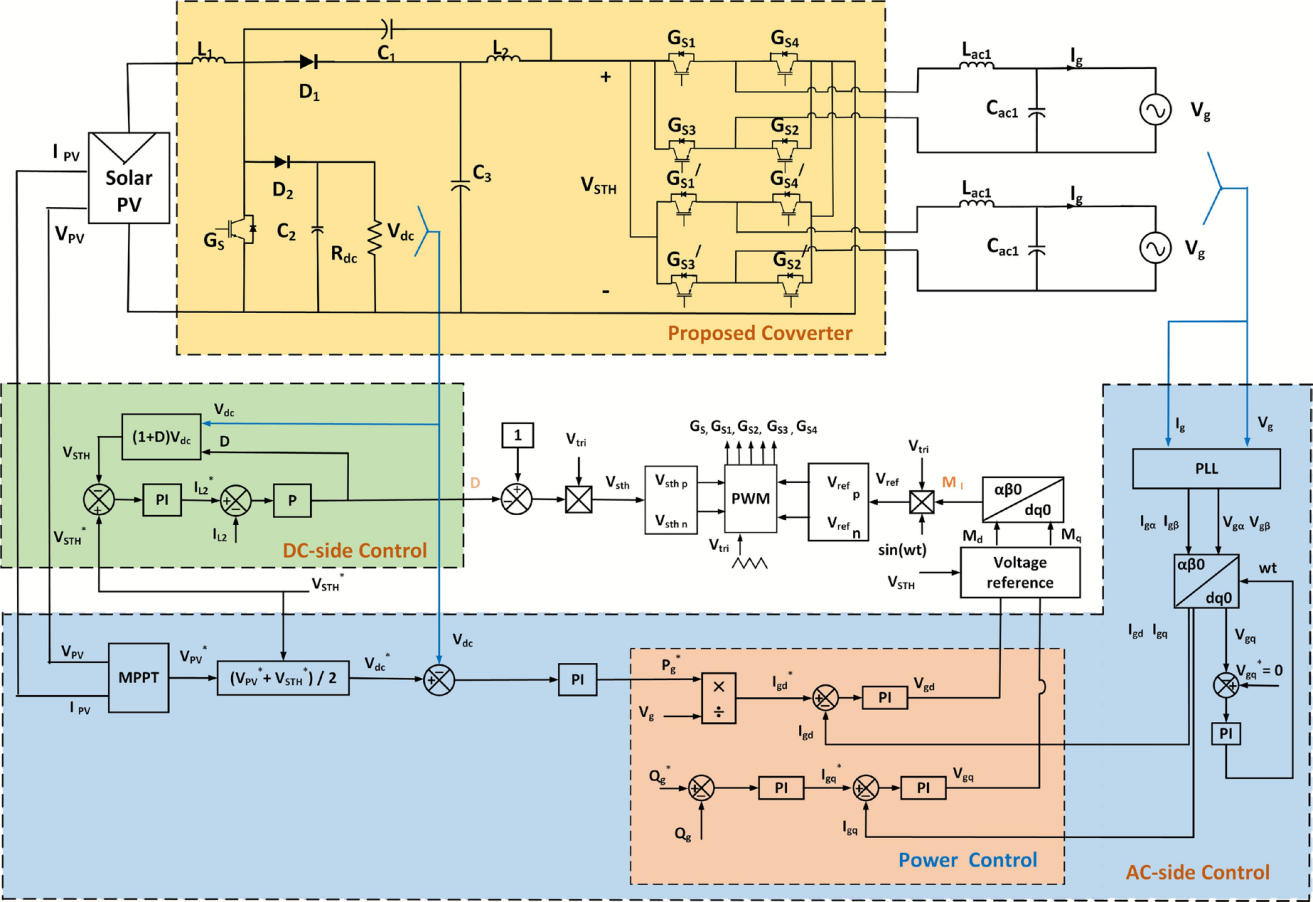
Overall configuration of presented solar PV hybrid multiple output system.

The overall layout of the presented solar PV microgrid system is illustrated in Fig. 2. The system consists of a solar PV array as the input source, a single-stage hybrid modified quasi-Z-source multi output converter (Q-ZSMOC), LC filter circuits, and coordinated dc-side and ac-side control units. Unlike conventional multi-stage architectures, the proposed configuration integrates voltage boosting, dc power extraction, and dc–ac conversion into a single power-processing stage. This enables the system to simultaneously supply one regulated dc output and multiple ac outputs without requiring an additional dc–dc converter or separate inverter stages.

The PV array feeds directly into the Q-ZSMOC system input, which ensures continuous input current and efficient power transfer. The boosted voltage of the dc-link generated by the converter is used to supply the dc load and to feed multiple single-phase inverter units. Each inverter output is connected to an LC filter to reduce switching harmonics before interfacing with ac loads or the utility grid. For control and regulation, the system implements a MPPT controller to draw maximum available power from the PV array and closed-loop voltage and power controllers to regulate the dc and ac outputs. A hybrid PWM strategy based on shoot-through control, sinusoidal pulse-width modulation and modulation index adjustment is employed to ensure proper switching and voltage boosting. Grid synchronization and reference signal generation are achieved using a phase-locked loop (PLL) and $\alpha \beta /dq$ transformations.

## Proposed converter topology description

The proposed Q-ZSMOC system is designed to obtain enhanced voltage gain and multi-output capability within a single-stage conversion structure. As viewed from the Fig. 2, the converter consists of three capacitors ($C_1$, $C_2$, and $C_3$), two inductors ($L_1$ and $L_2$), two diodes ($D_1$ and $D_2$), one auxiliary active switch ($G_S$), and one or more single-phase H-bridge inverter modules. Each H-bridge inverter comprises four MOSFET switches ($G_{S1}$, $G_{S2}$, $G_{S3}$, and $G_{S4}$) and is supplied from a common boosted dc-link voltage.

As compared to the conventional quasi Z-source topology, the key modification in the proposed topology is the inclusion of an additional switched-capacitor branch formed by capacitor $C_2$, diode $D_2$, and switch $G_S$. This branch operates in coordination with the non-shoot-through and shoot-through modes of the converter to enhance the voltage boosting ability. This modification fundamentally distinguishes the proposed converter from existing multiport or hybrid quasi-Z-source architectures, which either provide only ac outputs, rely on cascaded stages, or lack an independently regulated dc output port within a single-stage configuration. During non-shoot-through intervals, the switched capacitor is charged, while during shoot-through intervals, the saved energy is released to increase the effective dc-link voltage. Inductors $L_1$ and $L_2$ provide energy buffering and provide input current continuously from the PV source, while capacitors $C_1$ and $C_3$ support voltage boosting and dc-link stabilization. The regulated dc output voltage is directly obtained across capacitor $C_3$, enabling simultaneous dc power delivery.

The boosted voltage of the dc-link is supplied to multiple single-phase inverter units connected in parallel at the dc side. Each inverter generates an independent ac output after LC filtering. In the proposed system, two parallel single-phase inverters ($n = 2$) are used for validation. However, the topology is inherently modular and can be extended by connecting *n* inverter modules in parallel or series to obtain multiple ac outputs or higher voltage and power ratings. This modular structure makes the proposed converter suitable for hybrid microgrid applications requiring concurrent dc and ac power delivery.

### Operating principle of the proposed converter

**Fig. 3:**
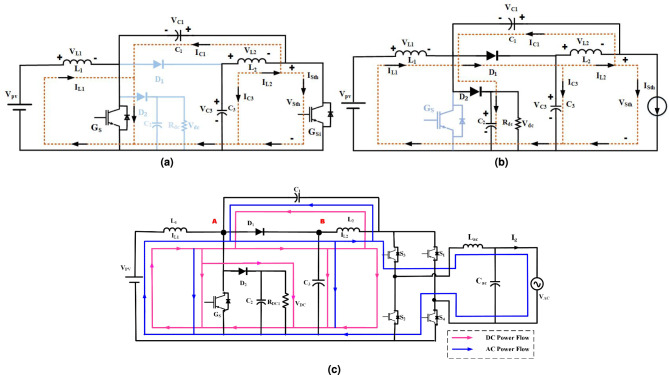
Operating waveform circuit of Q-ZSMOC in (**a**) shoot-through state. (**b**) Non-shoot-through state. (**c**) Power flow paths to loads.

The operation of the presented Q-ZSMOC in Fig. 3, is characterized by two distinct states of interval and corresponding power flow paths to differnt loads.: (i) Shoot-through interval ($DT_s$), (ii) Non-shoot-through interval ($(1-D)T_s$) and (iii) Power flow paths to dc and ac loads.

(i) Shoot-through interval ($DT_s$): Fig. 3(a) gives the equivalent circuit diagram of the presented converter’s shoot through interval. The diodes $D_1$ and $D_2$ get reverse biased and cease to conduct during this time. Concurrently, the capacitors $C_1$ and $C_2$ allow the inductor currents $I_{L_1}$ and $I_{L_2}$ to gradually charge up to their maximum levels. $G_{S_i}$ ($G_{S_1}- G_{S_4}$) or ($G_{S_2}$- $G_{S_3}$) shoots through the legs of the inverter bridge. Kirchhoff’s Voltage Law (KVL) is used to determine the capacitor-currents and inductor-voltages for the shoot-through interval from Fig. 3(a) as follows:

1 $$\begin{aligned} & V_{L_1} = V_{pv} \end{aligned}$$

2 $$\begin{aligned} & V_{L_1} = -V_{C_1} + V_{C_3} \end{aligned}$$

3 $$\begin{aligned} & V_{STH} = -V_{L_2} + V_{C_3} \end{aligned}$$

4 $$\begin{aligned} & I_{C_1} = -I_{L_1} - I_{C_3} - I_{STH} \end{aligned}$$

5 $$\begin{aligned} & I_{C_3} = -I_{L_2} \end{aligned}$$

6 $$\begin{aligned} & I_{C_1} = -I_{L_1} + I_{L_2} - I_{STH} \end{aligned}$$

(ii) Non-shoot-through state ($(1-D)T_s$): The suggested converter’s equivalent circuit diagram in non-shoot-through state is given in Fig. 3(b). The switch $G_S$ is off for this particular period, while the switch $G_{S_i}$ is in a power interval. Either the ($G_{S_1}-G_{S_4}$) or ($G_{S_2}-G_{S_3}$) inverter switches are conducting in this state of operation. Switch $G_S$ is made off at this time, which causes diodes $D_1$ and $D_2$ to become forward biased and conduct. Discharging of the inductors $L_1$ and $L_2$ happens simultaneously. Using Kirchhoff’s Voltage Law (KVL), the capacitor currents and inductor voltages during the non-shoot-through interval can be obtained from Fig. 3(b).

7 $$\begin{aligned} & V_{L_1} = V_{pv} - V_{C_2} \end{aligned}$$

8 $$\begin{aligned} & V_{C_2} = V_{C_3} \end{aligned}$$

9 $$\begin{aligned} & V_{L_2} = -V_{C_1} \end{aligned}$$

10 $$\begin{aligned} & V_{STH} = V_{C_3} + V_{C_1} \end{aligned}$$

11 $$\begin{aligned} & I_{C_1} = I_{L_2} - I_{STH} \end{aligned}$$

12 $$\begin{aligned} & I_{C_2} = I_{L_1} - I_{STH} - I_{C_3} \end{aligned}$$

13 $$\begin{aligned} & I_{C_3} = I_{L_1} - I_{STH} - I_{C_2} \end{aligned}$$

14 $$\begin{aligned} & I_{C_3} = -I_{C_1} - I_{STH} \end{aligned}$$

(iii) Power flow paths to dc and ac loads: The net power transfer paths of the presented Q-ZSMOC in the non-shoot-through interval is illustrated in Fig. 3(c). The net power delivered to the dc load is transferred from the PV source through the inductor $L_1$, node A, diode $D_2$, and is delivered to the dc-link capacitor $C_2$, from which the regulated dc voltage is obtained across the dc load. This power transfer path is highlighted using pink arrows in Fig. 3(c). Simultaneously, the ac load receives power from the same boosted dc-link through a parallel path. The net power transfer to the ac load follows the path from the PV source through $L_1$, node A, diode $D_1$, node B, and output inductor $L_2$, before being processed by the inverter bridge stage to generate the required ac output during power interval. This power flow is indicated using blue arrows in Fig. 3(c).

In normal operating states, the prsesnted Q-ZSMOC manages the dc-link voltage and ac output using fixed reference values of modulation index and duty ratio. When the PV input voltage decreases, the shoot-through duty ratio is increased to enhance the boost factor, allowing continuous and regulated power delivery to both dc and ac loads. Under load varrying conditions, the impedance network provides temporary energy buffering, while the controller dynamically adjusts the modulation index and duty ratio to maintain power balance and voltage stability between the outputs.

### Steady-state analysis of the presented system

From the volt–second balance^30^ applied to inductor $L_1$ over a switching period,:

15 $$\begin{aligned} & D V_{pv} + (1 - D)(V_{pv} - V_{C_2}) = 0 \end{aligned}$$

16 $$\begin{aligned} & V_{pv} - (1 - D) V_{C_2} = 0 \end{aligned}$$

From the volt–second balance applied to inductor $L_2$ over a switching period,:

17 $$\begin{aligned} & D(-V_{C_1} + V_{C_3}) + (1 - D)(-V_{C_1}) = 0 \end{aligned}$$

18 $$\begin{aligned} & -V_{C_1} + D V_{C_3} = 0 \end{aligned}$$

19 $$\begin{aligned} & -V_{C_1} + D V_{C_2} = 0 \end{aligned}$$

From Eq. (16),

20 $$\begin{aligned} V_{C_2} = \left( \frac{1}{1 - D} \right) V_{pv} \end{aligned}$$

From Eq. (8),

21 $$\begin{aligned} V_{C_3} = \left( \frac{1}{1 - D} \right) V_{pv} \end{aligned}$$

Put Eq. (20) in Eq. (19),

22 $$\begin{aligned} V_{C_1} = \left( \frac{D}{1 - D} \right) V_{pv} \end{aligned}$$

The dc-link average voltage, $V_{STH}$, at the input side of the inverter can be equated through Eqs. (10), (21), and (22) as follows:

23 $$\begin{aligned} & V_{STH} = \left( \frac{1 + D}{1 - D} \right) V_{pv} \end{aligned}$$

24 $$\begin{aligned} & V_{dc} = V_{C_2} = \left( \frac{1}{1 - D} \right) V_{pv} = B V_{pv} \end{aligned}$$

Where, $V_{dc}$ denotes the peak dc output voltage of the Q-ZSMOC and the corresponding dc output boost factor is given by,

25 $$\begin{aligned} B_f = \frac{V_{dc}}{V_{pv}} = \frac{1}{1 - D} \end{aligned}$$

It is to be noted that the simple boost PWM techniques is used for the presented system and is constrained by the following relation between the duty cycle (*D*) and the modulation index ($M_I$):

26 $$\begin{aligned} D + M_I \le 1 \end{aligned}$$

From Eq. (23), the ac peak voltage gain of the Q-ZSMOC outputs is obtained in terms of $M_I$ (where $M_I \le 1-D$) as

27 $$\begin{aligned} V_{ac_{pk1}} = V_{ac_{pk2}} = M_I V_{STH} = M_I \left( \frac{1 + D}{1 - D} \right) V_{pv} \end{aligned}$$

Here, $V_{ac_{pk}}$ ($V_{ac_{pk}} = V_{ac_{pk1}} = V_{ac_{pk2}}$) represents the ac peak voltage, and the ratio between the ac peak voltage and the input voltage is given by

28 $$\begin{aligned} & \frac{V_{ac_{pk}}}{V_{pv}} = M_I \left( \frac{1 + D}{1 - D} \right) \end{aligned}$$

29 $$\begin{aligned} & V_{ac_{pk1}} = V_{ac_{pk2}} = M_I (1 + D) V_{dc} \end{aligned}$$

## Comparative performance analysis

A comparative analysis highlighting the the main characteristics and constraints of conventional PV-fed Voltage Source Inverter-based (PV-VSI) converters, quasi-Z-source-based inverters, and the presented multi-output quasi-Z-source converter is summarized in Table 1.

**Table 1:** Qualitative comparison of conventional and proposed converter architectures.

Performance criteria	Conventional PV-fed VSI ^36^	Conventional hybrid system^37^	Proposed system
Number of outputs	Single AC output	Single DC output + one AC output (simultaneous)	Two independent AC outputs + one DC output
Conversion stages	Two-stage	Single-stage	Single-stage
Component count for multi-output implementation	High (additional DC–DC converters and inverter stages required)	Moderate (single boost + H-bridge supplies both DC and AC)	Reduced due to unified multi-output structure
DC voltage gain (*B*)	$B = \dfrac{V_{dc}}{V_{PV}}$ (no inherent boost)	$B = \dfrac{1}{1-D}$	$B = \dfrac{1}{1-D}$
AC voltage gain (*G*)	$G = M \cdot B$	$G = M_i \cdot \dfrac{1+D}{1-D}$	$G_{ac} = M_i \left( \dfrac{1 + D}{1 - D}\right)$, independently controlled
Switch voltage stress	$V_{sw} \approx V_{dc}$	$V_{sw} \approx \dfrac{V_{dc}}{1-D}$	Comparable stress level with redistribution across multiple energy-storage elements
Independent output voltage regulation	Not supported	Supported (coordinated duty cycle and PWM)	Fully supported for AC and DC outputs
Dynamic response to load variation	Moderate (affected by DC-link dynamics and cascaded control)	Moderate-fast (coordinated control of DC and AC outputs)	Fast and stable due to unified control structure
Key advantages	Mature and well-established topology Simple control implementation Wide industrial acceptance Well-understood industrial design practices	Single-stage hybrid DC–AC operation Simultaneous DC and AC output from a single DC source Reduced component count compared to cascaded systems	Single-stage hybrid multi-output operation Independent regulation of DC and AC outputs Continuous input current with reduced ripple, suitable for PV sources Explicit separation of control objectives Enhanced controllability within PWM limits
Key limitations	Requires additional DC–DC stage for voltage boost Increased component count and losses Discontinuous input current leading to higher ripple and EMI	Limited AC voltage amplitude due to H-bridge PWM Duty cycle and modulation index constrained by simultaneous DC/AC operation Requires coordination of control loops for stable operation	Duty-cycle and modulation index constrained by PWM limits Increased control design complexity due to multi-output regulation Input current ripple depends on impedance-network and capacitor sizing Requires careful coordination of multiple control loops

To ensure a fair and meaningful comparison, representative converter structures commonly reported in the literature are surveyed, including two-stage boost VSI configurations and single-stage Q-ZSI-based topologies capable of supplying dc or ac outputs. The comparison focuses on important performance aspects such as conversion stages, number of power outputs, voltage gain characteristics, switch stress behavior, dynamic response to load variations, output voltage regulation capability and ac output. It is observed that conventional VSI-based systems generally require multiple power conversion stages to achieve voltage boosting and multi-output functionality, whereas qZSI-based converters offer inherent voltage boost and continuous input current but are typically limited to single-output operation with coupled gain characteristics. In contrast, the proposed converter integrates multiple energy ports within a single-stage architecture, enabling independent regulation of multiple ac and a dc output without the need for additional power conversion stages. From Table 1, it is clearly evident that the presented topology preserves the core benefits of the conventional structure of quasi Z-source-based structures while extending their functional capability toward multi-output PV-fed applications, with performance characteristics comparable to conventional approaches under similar operating conditions.

## Control strategy of the presented solar PV system

The control structure of the single-phase photovoltaic Q-ZSMOC system, as depicted in Fig. 2, is divided into three subsections. The first subsection explains the decoupling analysis of duty ratio and modulation index, second subsection explains how the perturb and observe (P&O) method achieves the maximum power from the solar PV system. The third subsection describes how the power extracted from the PV module is converted from dc to ac and injected into the grid. This is achieved using two different control loops, consisting of an inner loop of current control that regulates the grid current and ensures power factor control, along with an outer loop of voltage control that maintains the dc capacitor voltages and governs the power delivered to the grid.

### Decoupling analysis of duty ratio and modulation index

On the basis of the steady-state performance evaluation of the proposed Q-ZSMOC, it is evident that the shoot-through duty ratio *D* and the modulation index $M_I$ influence different electrical variables of the system. The voltage of the dc-link derived in (24) is expressed as

30 $$\begin{aligned} V_{dc} = \frac{1}{1-D} V_{pv} \end{aligned}$$

where $V_{dc}$ represents the voltage of the dc-link across capacitor $C_2$ and $V_{pv}$ denotes the PV array voltage. Equation (30) indicates that, for a given PV array voltage, the dc-side voltage is solely governed by *D*.

The peak ac output voltage of the inverter can be listed from Eq. (29), is given by

31 $$\begin{aligned} V_{ac,pk} = M_I (1+D) V_{dc} \end{aligned}$$

where $V_{ac,pk}$ is the peak ac output voltage. Eq. (31) shows that the magnitude of the ac-side voltage can be independently managed by the $M_I$ once the dc-link voltage is regulated. The PWM operating constraint defined in Eq. (26) specifies the feasible operating region of the converter. Within this region, The $M_I$ and the *D* are independently regulated control variables, as the constraint does not introduce dynamic coupling between the two control parameters.

Furthermore, decoupling is reinforced through time-scale separation in the control structure. The *D* is adjusted by the MPPT controller, which operates at a slower time scale dictated by irradiance and temperature variations. In contrast, the modulation index $M_I$ is regulated by the grid-side current controller synchronized with the utility grid and operates at a significantly higher bandwidth. Consequently, under dynamic irradiance conditions, variations in *D* primarily affect dc-link voltage, while regulation of $M_I$ the compensates fast inner loop control of current, thereby preserving independent control of dc and ac outputs.

In conventional quasi-Z-source inverter control, *D* is commonly employed as a single dominant control variable, which inherently couples ac output regulation and the dc-link voltage boosting. While such an approach is suitable for single-output inverter systems, it limits flexibility in hybrid multi-output configurations where simultaneous and independent regulation of dc and ac ports is required. In contrast, the proposed control framework exploits the structural characteristics of the modified Q-ZSMOC to distinctly assign dc-side voltage regulation to the *D* and ac-side voltage regulation to the modulation index. This explicit separation of control objectives alleviates the inherent coupling associated with classical shoot-through-based qZSI control and enables improved controllability of hybrid outputs within the defined PWM operating region.

The closed-loop behavior of the proposed multi-output control scheme, including stability and interaction between outputs, can be described using conventional PI-based control laws. The shoot-through duty ratio *D*(s), which regulates the impedance-network energy and dc output voltage, is expressed as

32 $$\begin{aligned} D(s) = K_{p,dc} \, (V_{dc}^* - V_{dc}) + K_{i,dc} \, \frac{1}{s} (V_{dc}^* - V_{dc}) \end{aligned}$$

where $V_{dc}^*$ is the reference dc bus voltage, $V_{dc}$ is the measured dc bus voltage, $K_{p,dc}$ is the proportional gain of the dc voltage loop, $K_{i,dc}$ is the integral gain of the dc voltage loop, and *s* is the Laplace operator.

Similarly, the ac-side modulation index $M_I$, responsible for inverter voltage synthesis, is obtained from the ac voltage regulation loop as

33 $$\begin{aligned} M_I(s) = K_{p,ac} \, (V_{ac}^* - V_{ac}) + K_{i,ac} \, \frac{1}{s} (V_{ac}^* - V_{ac}) \end{aligned}$$

where $V_{ac}^*$ and $V_{ac}$ are the reference and measured ac voltages, respectively, and $K_{p,ac}$ and $K_{i,ac}$ are the proportional and integral gains of the ac voltage loop.

Since *D* and $M_I$ act on physically distinct energy-processing stages, dynamic interaction among outputs is inherently limited. Stable operation is ensured as long as the practical PWM constraint equation (26) is satisfied, which defines the feasible operating region of the presented converter.

### MPPT control strategy for the PV system

The proposed PV system utilizes the perturb and observe (P&O) control technique due to its simplicity of operation and ease of implementation. The output current and voltage of the PV module, $I_{pv}$ and $V_{pv}$ are used to compute the instantaneous power output $P_{pv}$. The P&O algorithm compares the present voltage and power values $V_{pv}(i)$ and $P_{pv}(i)$ with their previous values $V_{pv}(i-1)$ and $P_{pv}(i-1)$, respectively, to determine whether the PV operating voltage should be increased or decreased.

This iterative process continues until the difference between successive power samples approaches zero, ensuring operation at the maximum power point. Accordingly, the reference PV voltage is continuously updated, resulting in a corresponding shoot-through duty ratio reference $D^*$. As the operating point moves along the maximum power point curve, the PV array voltage is repeatedly adjusted based on the direction of power variation.

Thus, the MPPT controller receives the PV voltage and current as inputs and produces a reference duty cycle. This duty cycle is compared against a high-frequency triangular waveform $V_{tri}$, while two shoot-through signals $V_{sth,p}$ and $V_{sth,n}$ are employed as inputs for PWM generation. Consequently, the voltage of the dc-link is boosted in accordance with the shoot-through intervals.

According to the operating modes of the presented converter, some basic relationships can be listed from Eqs. (23), (24) and (28) as follows,

34 $$\begin{aligned} {\left\{ \begin{array}{ll} V_{C2} = V_{dc} = \dfrac{1}{1-D} V_{pv} = B_f V_{pv} \\ V_{ac,pk} = M_I V_{STH} = M_I (1+D) V_{dc} \end{array}\right. } \end{aligned}$$

where *T* denotes the switching period, $D = T_{ST}/T$ is the shoot-through duty ratio and $T_{ST}$ represents the shoot-through time interval within one switching cycle. $V_{dc}$ and $V_{ac,pk}$ are the peak ac and dc output voltages. $B_f$ is the dc voltage boosting factor, and $M_I$ is the modulation index. From (34), it can be concluded that the boosting of the dc-link voltage is mainly governed *D*.

#### Interaction of P&O MPPT with Q-ZSMOC internal dynamics

The shoot-through operation and energy exchange within the impedance network in quasi-Z-source–based converters introduces inherent low-frequency power ripple in the dc link. If directly reflected at the PV terminals, such ripple can interfere with P&O MPPT algorithms by causing incorrect perturbation decisions. This interaction becomes particularly relevant in hybrid converter topologies, where the dc-link dynamics and inverter operation are tightly coupled and may influence the PV-side operating conditions.

In the proposed Q-ZSMOC system, the interaction between internal converter dynamics and MPPT behaviour is effectively mitigated through control time-scale separation and the inherent filtering properties of the quasi-Z-source impedance network. The P&O MPPT algorithm operates at a significantly reduced update rate in comparison to the switching frequency and the inner voltage and current control loops. Consequently, high-frequency switching ripple and second-harmonic power oscillations introduced by the shoot-through states are averaged out before being processed by the MPPT controller.

Moreover, the capacitors and inductors of the quasi-Z-source impedance network inherently act as a low-pass filter linking the PV array and the inverter stage, attenuating high-frequency ripple components in the measured PV voltage and current. As a result, the P&O MPPT controller primarily operates on the averaged PV power rather than instantaneous oscillatory values. Additionally, the shoot-through duty ratio is regulated exclusively by the MPPT loop, whereas the modulation index is controlled independently by the fast grid-side current controller. This hierarchical control structure prevents short-term inverter-induced power fluctuations from propagating into the MPPT loop and ensures that MPPT dynamics do not adversely affect dc-link voltage regulation or ac output voltage stability. Hence, reliable and stable maximum power point tracking is achieved even under dynamic irradiance conditions, not withstanding the inherent ripple characteristics of quasi-Z-source converters. The P&O algorithm is selected in this work due to its simple implementation, low computational burden, and proven robustness in real-time photovoltaic applications, making it well suited for practical implementation and hardware-in-the-loop validation.

### Grid-side power control of the PV system

The closed loop controller of the grid-connected PV system plays a vital role in regulating the outputs to their desired values subject to dynamic load variation of input and intended to transform dc power to ac power for grid injection with unity power factor and grid current requirements. With the PV panel MPPT controller, the electrical power supplied by the PV array to the ac loads and grid are adjusts the output power by the closed-loop control where the ac power output is approximately equal to the PV panel output power that is $P_{out} \approx P_{pv}$, expressed as

35 $$\begin{aligned} P_{out} = V_g I_g \cos (\omega t) \end{aligned}$$

Where ‘$I_g$’ and ‘$V_g$’ are the current and voltage of the utility grid and *cos*(*wt*) is the power factor.

In a grid-connected PV system, the grid voltage and power factor are already known. According to equation (31), it can be assumed that the relationship between the grid current and output power is proportional. This means that as the grid current is increased, the output power generated by the PV module also increases. For the regulation of PV module output power, a closed-loop control mechanism is employed. This control involves two separate loops, an outer loop for voltage control and an inner loop for current control. The dc-bus voltage is controlled by the outer voltage loop controls while the inner loop of current control is used for controlling the current and obtaining unity power factor. When the load fluctuates, the capacitor voltages of the Q-ZSIs may drop excessively; in such scenario, $M_I$ is incapable of recovering the desired ac output. This will inject grid current harmonic content. Thus, the voltage control outer loop is used for regulating the dc-bus voltage and stabilizing the capacitor voltages of the Q-ZSIs ensuring that the the grid-injected current from the inverter is controlled to remain in phase with the grid voltage and the inverter voltages could match up with the ac grid voltage. The entire control structure of the grid-connected Q-ZSMOC system is illustrated in Fig. 2 which is divided into dc-side control and grid-side inverter control. The dc-side controller uses a Proportional-Integral (PI) controller to adjust the dc-bus voltage ‘$V_{STH}$’ by providing with a fixed average dc-link voltage ‘${V_{STH}}^*$’ to the inverter. Further, another P controller is used to assure that the peak voltage of the dc-link is kept at the desired level, and thus the desired *D* is achieved, which regulates the dc-bus voltage as well as the dc capacitor voltages despite variations in the load. From Eq. (30), it can be derived that,

36 $$\begin{aligned} V_{STH} = (1 + D) V_{dc} \end{aligned}$$

In contrast, a closed loop power control technique is employed for grid-side control, which involves transforming a synchronous d-q reference frame and controlling the transmitted power to the grid in terms of set point of reference. In order to do this, the controller is designed so that the orthogonal stationary $\alpha$-$\beta$ components are produced from the signals after the control loop detects the grid voltage and current. Then, with the use of a single-phase locked loop (PLL), the $\alpha$-$\beta$ components are converted into d-q components. It can be inferred from Eqs. (32) and (34) that,

37 $$\begin{aligned} V_{C_2} = V_{dc} = \frac{1}{2} (V_{pv} + V_{STH}) \end{aligned}$$

Thus, from the above equation, the reference dc voltage $V_{dc}^*$ could be set by the PV array voltage reference produced by the MPPT module, and required peak dc-link voltage $V_{STH}$.

Since output power of the PV array and capacitor voltages are directly correlated, controlling capacitor voltages will also allow for output power adjustment. A PI controller is used for this purpose. The current reference is obtained by comparing the capacitor voltage of $V_{C_2}$ (or $V_{dc}$) with a reference value $V_{dc}^*$ . The peak value of the direct-axis reference current $I_{gd}^{*}$ supplied to the grid can be expressed as:

38 $$\begin{aligned} I_{gd}^* = \frac{P_{g}^*}{V_g} \end{aligned}$$

where $P_{g}^*$ is the active power reference generated by the MPPT controller and $V_g$ denotes the voltage of the grid. A PI controller is employed to manage the reference $q$-axis grid current $I_{gq}^{*}$. Subsequently, for the control the quadratic component of the measured grid current $I_{gq}$, another PI controller is used to adjust the q-component of the grid voltage $V_g$ . Thus, the d and q-components of the modulation index $M_d$ and $M_q$ can be obtained which are further transformed from *d*-*q* to $\alpha$-$\beta$ components. Hence, with the combination of the $M_I$ and *D*, the gate signals are produced for the switching of the IGBTs of the Q-ZSMOC system. From (24) and (27), the dc output power ($P_{dc}$) and ac output power ($P_{ac}$=$P_{{ac}_1}$=$P_{{ac}_2}$) can be deduced as:

39 $$\begin{aligned} \left\{ \begin{aligned} P_{dc}&= \frac{V_{dc}^2}{R_{dc}} = \frac{V_{pv}^2}{R_{dc}(1-D)^2} \\ P_{{ac}_1}&= \frac{V_{{ac}_1}^2}{R_{ac_1}} = \frac{V_{ac_{pk1}}^2}{2R_{ac_1}} = \frac{M_I^2 V_{pv}^2(1+D)}{2R_{ac_1}(1-D)^2} \\ P_{{ac}_2}&= \frac{V_{{ac}_2}^2}{R_{ac_2}} = \frac{V_{ac_{pk2}}^2}{2R_{ac_2}} = \frac{M_I^2 V_{pv}^2(1+D)}{2R_{ac_2}(1-D)^2} \end{aligned} \right. \end{aligned}$$

From (39), it can be seen that $P_{dc}$ is only dependent on *D* and $P_{ac}$ is dependent on *D* as well as $M_I$. The minimum requirement that needs to be met by $M_I$ and *D* is given in Eq. (26). The presented converter outputs are governed by Eqs. (24), (27) and (36). Looking at the voltage expressions, It is clear that the dc voltage $V_{dc}$ is determined by *D*, whereas the peak ac voltage $V_{ac_{pk}}$ is influenced by both $M_I$ and *D*. Therefore, ’$V_{dc}$’ can be controlled by *D* and ’$V_{ac_{pk}}$’ can be controlled by $M_I$ only, since *D* should be maintained at a certain level so that it does not create disturbance in ’$V_{dc}$’ output and regulating ’$V_{STH}$’ at the same time by comparing it with a command value. Thus, decoupled control of the output voltages can be obtained by using $M_I$ and *D*, subject to (26) holding true and maintaining a fixed ’$V_{STH}$’ for desired values of output. It is noted that the proposed decoupling is valid within the PWM constraint as given in (26), which defines the feasible operating region of the converter. Within this region, the proposed control strategy achieves effective independent regulation of the dc and ac outputs.This guarantees the independent regulation of each output in respect of the others. The duty cycle *D* from the MPPT and two shoot-through voltages (+$V_{sth}$ and -$V_{sth}$) are compared with a switching frequency 10kHz triangular waveform ($V_{tri}$) for the generation of shoot-through states and two reference sinusoidal signals with similar frequencies of 50 Hz and peak amplitude of +$V_{ref}$ and -$V_{ref}$ which are both equal in amplitude and $180^0$ displaced in phase are used as inputs for the PWM generation.

**Table 2:** System parameters.

Parameter	Value
Total capacity of PV system	16 kW
Open-circuit voltage	48.6 V
Short-circuit current	6.3 A
Voltage at $P_{MPP}$	40.5 V
Current at $P_{MPP}$	5.93 A
Maximum power of PV array ($P_{MPP}$)	240.16 W
Number of strings in parallel	9
Number of modules per string	8
Reference temperature ($T_{ref}$)	$25^{\circ }\textrm{C}$
Reference illumination ($I_{ref}$)	$1000~\mathrm {W/m^2}$

**Fig. 4:**
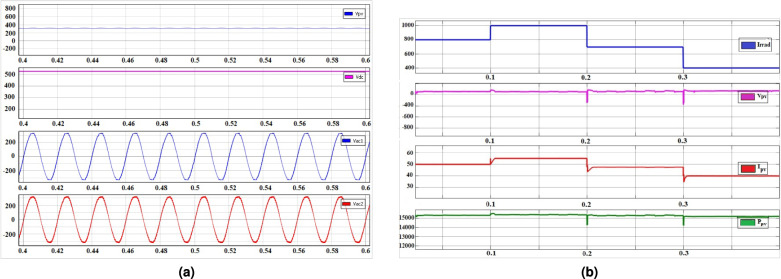
(**a**) Steady state output voltages of dc and ac with corresponding input voltage of solar PV, (**b**) PV outputs dynamic response with step change in illumination.

**Fig. 5:**
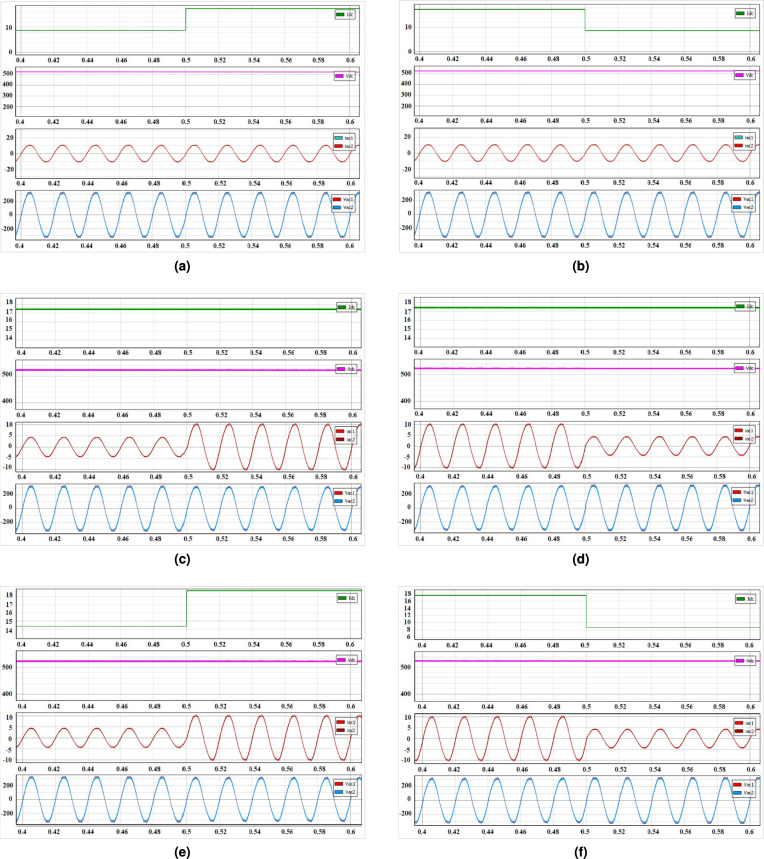
Closed-loop dynamics of the presented system showing ac and dc output voltages with (**a**) stepping-up of dc load current, (**b**) dc load current stepped-down, (**c**) ac1 and ac2 load currents stepped-up, (**d**) ac1 and ac2 load currents stepped-down, (**e**) both ac1, ac2 and dc load currents stepped-up, (**f**) both ac1, ac2 and dc load currents stepped-down.

**Fig. 6:**
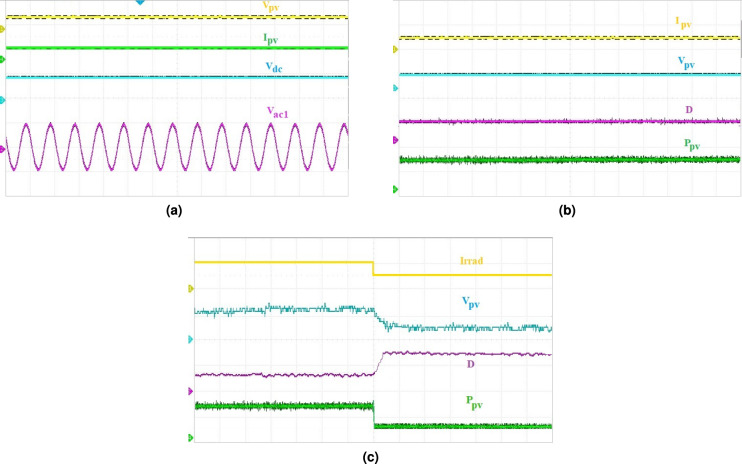
(**a**) Steady-state output voltages, where ($V_{dc}$ = 500 V/div, $V_{ac_{1}}$ = $V_{ac_{2}}$ = 300 V/div), corresponding to PV inputs $V_{pv}$ = 500 V/div and $I_{pv}$ = 100 A/div and time = 20 ms/div, (**b**) steady-state PV inputs, where ($I_{pv}$ = 100 A/div, $V_{pv}$ = 500 V/div, $P_{pv}$ = 1000 W/div) corresponding to duty ratio (D = 0.5/div) and time = 20 ms/div, (**c**) PV panel output voltage and power, where ($V_{pv}$ = 300 V/div and $P_{pv}$ = 1000 W/div) corresponding to change in irradiation (Irrad = $1000W/m^2$/div), duty ratio (D = 0.5/div) and time = 20 ms/div.

## Simulation verification

### Steady-state verification of the presented system

To validate the proposed system, simulation verification of the system is performed in MATLAB/Simulink using a discrete-time simulation environment. The simulations are carried out with a $f_s = 10$ kHz switching frequency of, a control sampling time of $T_s = 5~\upmu$s, and a fixed-step discrete solver to accurately capture the control behavior and switching dynamics of the presented converter. The hybrid multi-output converter as proposed provides one boost dc and dual ac outputs having a total capacity of 16 kW that is applicable to fulfil a small residential house. The simulation model uses SUNPOWER SPR 240E-WHT-D PV panel which comprises of 8 PV modules connected in series in each string and 9 parallel strings. Each module consists of a total of 72 cells. The PV panel design specification of the parameters are listed with their values in Table 2. It is to be noted that the values of maximum PV array power, current and voltage at maximum power, open-circuit voltage and short-circuit current as mentioned in Table 2 are for one parallel and series string. Fig. 4(a) illustrates the steady-state prformance of the dc and ac outputs with the corresponding values of PV input. The simulation results for the presented system are obtained for $D = 0.4$ and $M_I = 0.45$. It is seen that, for a input dc voltage of $V_{pv} = 312$ V, the presented system gives one dc boost output $V_{dc} = 520$ V and two ac boost outputs $V_{ac_1} = V_{ac_2} = 325$ V (peak) for a load of $30~\Omega$. The values of inductors are calculated as $L_1 = 5$ mH, $L_2 = 2$ mH and capacitors $C_1 = 60~\upmu$F, $C_2 = 760~\upmu$F, and $C_3 = 147~\upmu$F.

**Fig. 7:**
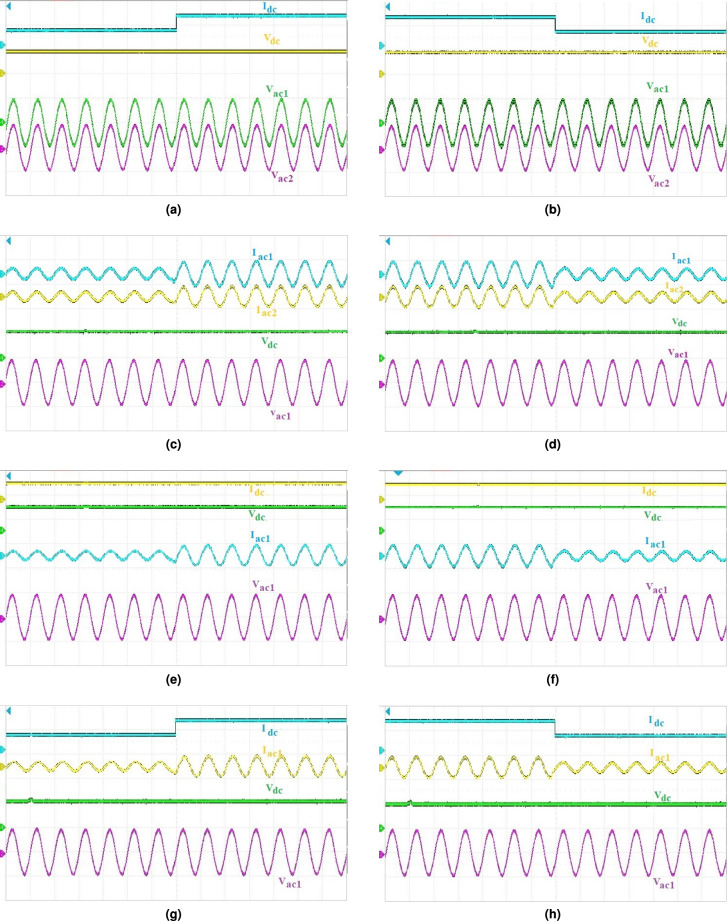
Dynamic performance of proposed system, where ($V_{dc_1}$ = 500 V/div, $V_{ac_1}$ = $V_{ac_2}$ = 300 V/div), with (**a**) step-up change in $I_{dc_1}$, where ($I_{dc_1}$ = 10 A/div), (**b**) step-down change in $I_{dc_1}$, where ($I_{dc_1}$ = 10 A/div), (**c**) step-up change in $I_{ac_1}$ and $I_{ac_2}$, where ($I_{ac_1}$ = $I_{ac_2}$ = 10 A/div), (**d**) step-down change in $I_{ac_1}$ and $I_{ac_2}$, where ($I_{ac_1}$ = $I_{ac_2}$ = 10 A/div), (**e**) step-up change in $I_{ac_1}$ only ($I_{ac_1}$ = 10 A/div), (f) Step-down change in $I_{ac_1}$ only, ($I_{ac_1}$ = 10 A/div), (**g**) step-up change in both $I_{dc_1}$ and $I_{a_{c_1}}$, $I_{ac_{1}}$, $I_{ac_{2}}$, where ($I_{ac_1}$ = $I_{ac_2}$ = 10 A/div), (**h**) step-down change in both $I_{dc_1}$ and $I_{ac_1}$, $I_{ac_{2}}$, where ($I_{ac_1}$ = $I_{ac_2}$ = 10 A/div) and time = 20 ms/div.

### Dynamic performance of the presented system

The dynamic performance of the presented PV system is evaluated using simulation results as shown in Fig. 4(b). It is performed with step changes in irradiation (G) of 800 $W/m^2$, 1000 $W/m^2$, 700 $W/m^2$, 400 $W/m^2$ and fixed temperature (T) of $25^0C$, in order to check the behavior of voltage ‘$V_{pv}$’, current ‘$I_{pv}$’ and power ‘$P_{pv}$’ with the MPPT controller. Closed-loop control is implemented to validate the system’s dynamic response and the simulated results are illustrated in Fig. 5. The dynamic analysis of the presented system is carried out by firstly stepping up the dc side load ‘$I_{dc_1}$’ from 8.6 A to 17.3 A, with ‘$R_{dc_1}$’ is decresed from 60 $\Omega$ to 30 $\Omega$ keeping the ac side loads unchanged and the corresponding results of dc and ac output voltages are observed as shown in Fig. 5(a). Similarly, the same step has been repeated by stepping down the dc side load ‘$I_{dc_1}$’ from 17.3 A to 8.6 A, with ‘$R_{dc_1}$’ increased from 30 $\Omega$ to 60 $\Omega$ and the results of which are illustrated in Fig. 5(b). Secondly, the ac side loads ‘$I_{ac_1}$’ and ‘$I_{ac_2}$’ are stepped up from 5.4 A to 10.8 A, with ‘$R_{dc_1}$’ and ‘$R_{dc_2}$’ reduced from 60 $\Omega$ to 30 $\Omega$ keeping the dc side load unchanged and the corresponding values of output dc and ac voltages are observed as shown in Fig. 5(c). Similarly, the same step has been repeated by stepping down the loads of ac side, results of which are illustrated in Fig. 5(d). Thirdly, both the dc side load ‘$I_{dc_1}$’ and ac side loads ‘$I_{ac_1}$’ and ‘$I_{ac_2}$’ are stepped up at the same time and the output results are shown in Fig. 5(e). Similar step has been carried out with the loads stepped down the results of which are given in Fig. 5(f). From above obtained results it is found that the output voltages in both dc and ac sides are well regulated and maintained at desired values with the proposed decoupled control technique and and is able to obtain an decoupled control of both outputs, provided the limiting conditions are satisfied, which verifies the proper functioning of the decoupled control strategy.

## Validation

### Steady-state results of the presented system

The proposed system is analyzed in real time using the Typhoon HIL-402 platform. The real time steady-state output results corresponding to PV inputs is shown in Fig. 6(a). For the limitation of output ports in digital oscilloscope, $V_{ac_2}$ which is equal in amplitude to $V_{ac_1}$ is not shown. Figure 6(b) shows the current, voltage and power output of the PV panel corresponding to duty ratio D. Figure 6(c) shows the output power with step change in irradiation. It is clear from the results that with the change in temperature or irradiation, the output voltage of the PV panel is continuously working to achieve the voltage at MPP with the shoot-through duty ratio updated accordingly in order to keep the peak dc-link voltage steady under the designed control operations.

### Dynamic behavior of the presented system

The real-time results of dynamic response in closed loop for the presented system is illustrated in Fig. 7. It can be observed that, when the dc load undergoes a $50\%$ step increase from the initial value of $I_{dc_1}$ from 8.6 A to 17.5 A, there is some loss while running it in real-time mode in the dc output and as such in the ac outputs as well. The values of output voltages in real-time mode are: $V_{dc_1}$ = 500 V and $V_{ac_1}$ = $V_{ac_2}$ = 305 V as shown in Fig. 7(a). Similar case is seen in all the real-time results of Fig. 7. Figure 7(b), depicts the output results with stepped-down of dc load ‘$I_{dc}$’. Secondly, Fig. 7(c) depicts the output results for stepped-up of ‘$I_{ac_1}$’ and ’$I_{ac_2}$’ and the same has been repeated by stepping down ‘$I_{ac_1}$’ and ‘$I_{ac_2}$’ as shown in Fig. 7(d). Figure 7(e), (f) depicts the results of step-up and step-down of one ac load keeping dc load constant. Lastly, results were obtained for step-up and step down of both ‘$I_{dc}$’and ‘$I_{ac_1}$’, ‘$I_{ac_2}$’ loads at the same time, as shown in Fig. 7(g), (h).

It is observed from the results that disturbances applied to one output port do not adversely affect the regulation of the remaining outputs. During dc-side load and irradiance variations, the ac output voltage and grid-side currents remain well regulated, while ac-side disturbances do not affect the dc-link or dc output voltage. This behavior confirms effective dynamic decoupling among the multiple outputs achieved by the proposed control strategy.

## Conclusion

This paper introduces a novel hybrid system integrating solar PV, which can provide controlled hybrid multiple outputs: one boosted dc and multiple ac power to the loads. For this, a Q-ZSMOC-based PV system is presented with detailed operation and steady-state analysis along with a control scheme. The supplied power by the converter can be utilized to serve a residential home load and fed into the microgrid as well. The proposed control helps in regulating the ac and dc bus voltages despite variations in the input conditions and obtain a desired power output for household loads and the utility grid. Simulation results were obtained to show the performance of MPPT under different values of irradiation and temperature and the presented PV system is tested with a hybrid load power control algorithm under various conditions of load. To validate the operation of the controllers, the regulation of output power with dynamic loading and an evaluation of the overall performance of the proposed PV hybrid system is conducted.

Although the proposed PV system connected Q-ZSMOC has been validated at the studied power level, extension to higher-power microgrid applications necessitates careful consideration of practical design aspects. At increased power ratings, thermal behavior of power semiconductor devices, voltage and current stresses on the quasi-Z-source impedance network components, and overall conversion efficiency become critical factors influencing system performance. The inherent single-stage power conversion structure and boosting ability of the quasi-Z-source network topology are favorable for scalable operation; however, appropriate device selection, thermal design, and passive component sizing are essential to ensure reliable performance at higher power levels.

## Data Availability

The datasets used and/or analysed during the current study are available from the corresponding author on reasonable request.
